# FABP4 suppresses proliferation and invasion of hepatocellular carcinoma cells and predicts a poor prognosis for hepatocellular carcinoma

**DOI:** 10.1002/cam4.1511

**Published:** 2018-05-07

**Authors:** Cheng‐Qian Zhong, Xiu‐Ping Zhang, Ning Ma, Er‐Bin Zhang, Jing‐Jing Li, Ya‐Bo Jiang, Yu‐Zhen Gao, Yan‐Mei Yuan, Shi‐Qian Lan, Dong Xie, Shu‐Qun Cheng

**Affiliations:** ^1^ Department of Hepatic Surgery VI Eastern Hepatobiliary Surgery Hospital Second Military Medical University Shanghai China; ^2^ LongYan First Hospital Affiliated to Fujian Medical University Fujian China; ^3^ Laboratory of Molecular Oncology Institute for Nutritional Sciences Shanghai Institutes for Biological Sciences University of Chinese Academy of Sciences Shanghai China; ^4^ Department of Molecular Diagnosis Clinical Medical School of YangZhou University Subei People's Hospital Yangzhou China

**Keywords:** Fatty acid‐binding protein 4, hepatocellular carcinoma, poor prognosis, proliferation and invasion

## Abstract

Adipocyte fatty acid‐binding protein (FABP4) is abundant in macrophage and adipocyte. It is known to be involved in lipid metabolism. The role of FABP4 has been reported in various cancers, such as non‐small cell lung cancer, breast cancer, ovarian cancer, and prostatic cancer. However, its role remains unclear in hepatocellular carcinoma (HCC). In our study, we investigated the expression of FABP4 at both mRNA and protein levels, and by examining 175 cases of patients with cancer of the liver tissue microarray, the significance between the expression of FABP4 and clinical characteristics had been discussed. We found that FABP4 was lowly expressed in HCC tissues compared to the corresponding tissue adjacent, and the expression of FABP4 was significantly associated with the tumor size, PVTT, recurrence‐free survival and overall survival. Moreover, multivariate Cox regression analysis indicated that the expression of FABP4, Alb, AFP, HBsAg, and PVTT were independent risk factors for overall survival, and the expression of FABP4, AFP, GGT, tumor size, and encapsulation were independent risk factors for HCC recurrence. In addition, we revealed that FABP4 suppressed HCC cell proliferation and invasion in vitro. Moreover, overexpression of FABP4 led to inhibit tumor growth and decreased tumor volume in vivo. These phenotypes were associated with altered expression of Snail and p‐STAT3. Our studies thus suggest that FABP4 could be a potential target for HCC chemotherapy.

## Introduction

Hepatocellular carcinoma (HCC) is one of the most common malignant tumors in the world. It is the fifth most commonly occurring cancer and the second largest cause of cancer mortality in the worldwide 1. More and more evidence revealed that HCC was associated with multi‐gene mutation 2. Although the therapy of HCC has made little progress at the early stage, however, most patients are at later stage, and there is no effective treatment for these patients 3. Molecular targets now as a novel therapy for advanced HCC patients, it acquired favorable curative effect and significant prolong the HCC patient's survival time 4. Multiple molecular pathways are investigated in the occurrence and development of HCC, such as vascular endothelial growth factor receptor(VEGFR), epidermal growth factor receptor (EGFR), extracellular signal‐regulated kinase 1/ 2(ERK1/2) and phosphoinositide 3‐kinase / protein kinase B(PI3K/AKT)pathway 5, 6, 7. Preliminary progress has been made in the molecular‐targeted therapy of HCC. Further research will focus on the diagnosis and prognosis biomarker in the early of HCC.

As a member of the FABP family, Adipocyte fatty acid‐binding protein 4 (FABP4) is expressed abundantly in macrophage and adipocyte. It specificity binds hydrophobic lipid ligands and regulates glucose and lipid metabolism, and plays a role in signal transduction 8, 9, 10, some studies also found that FABP4 could effected cell proliferation and apoptotic processes 11. The function of FABP4 has been observed in various cancers, including non‐small cell lung cancer, breast cancer, bladder cancer, ovarian cancer, and prostatic cancer 12, 13, 14, 15, 16, 17, however, its role in HCC remained unclear.

In our study, we investigated the expression of FABP4 in HCC from both mRNA and protein level. We demonstrated the significance of FABP4 in HCC cell proliferation and invasion. Furthermore, we found that FABP4 is low‐expression in human HCC tissues, and the expression level was significantly related to increased tumor sizes and poor prognostic. In vitro assays showed that FABP4 significantly suppressed HCC proliferation and migration. In vivo, overexpression of FABP4 led to slowing tumor growth and decreasing tumor weight.

## Materials and Methods

### HCC tissue samples

Two hundred and thirty‐nine pairs of primary HCC tissues and their corresponding adjacent normal tissues were obtained from patients who underwent hepatectomy between February 2002 and July 2012 at the Eastern Hepatobiliary Surgery Hospital (EHBH), Shanghai, China. All of them signed informed consent. Among these patients, 64 HCC patients were recruited between May 2010 and July 2012, the 50 of these 64 paired samples were subjected to RNA extraction for quantitative real‐time PCR (qRT‐PCR) analysis and the others 14 paired samples were applied to protein extraction for Western‐blot detection. A total of 175 HCC patients were recruited between February 2002 and June 2006 all of the patients met the following inclusion criteria and underwent tissue microarrays (TMA) analysis: (a) a definite clinically diagnosed with HCC and postoperative pathological diagnosis of HCC, (b) R0 resection of all patients based on histologic examinations, (c) complete clinicopathologic and follow‐up data, (d) without distant metastasis, (e) patients did not receive treatment before surgery received any treatment before surgery. Our study was approved by the Institutional Review Board of the Institute for Eastern Hepatobiliary Surgery Hospital.

### RNA preparation and real‐time PCR

Preparation of RNA and complementary DNA (cDNA) samples were performed as described earlier. The specimens were snap‐frozen in liquid nitrogen and stored at −80°C until analysis. The total RNA of tumor tissues and adjacent noncancerous tissues from the 50 patients was isolated using Trizol reagent (Invitrogen, Carlsbad, CA), and reverse‐transcribed to cDNA using the PrimeScript RT reagent Kit (DRR037A, Takara, Japan) according to the manufacturer's instructions. SYBR Premix Ex Taq (DRR081, Takara) was used for real‐time PCR. The FABP4‐specific primers were 5‐ CGGAA TTCAT GTGTG ATGCT TTTGT AGGTACC‐3′(forward) and 5′‐ATAAG AATGC GGCCG CTTAT GCTCT CTCAT AAACT CTCGTG‐3′ (reverse), and the GAPDH primers were 5′‐ATGAC CCCTT CATTG ACCTCA‐3′ (forward) and 5′‐GAGAT GATCA CCCTT TTGGCT‐3′(reverse). GABPD acted as the internal control. The relative mRNA level of target genes to that of GAPDH in clinical samples was calculated according to the methods described by Wang et al. 18.

### TMAs and IHC

The tissue microarray included tumor tissues and adjacent noncancerous tissues of 175 patients were constructed as previously described 19. The tissue microarray slides were stained using the semi‐quantitative system according to the manufacturer's instructions, with a rabbit polyclonal antibody (Abcam, Kendall Square, Cambridge, MA, USA, ab13979, 1:100). Multispectral images (8 bit) acquired by the Vectra platform (Perkin‐Elmer, Waltham, MA) were processed by Nuance 30.0 software (Perkin‐Elmer) to build unique spectral curves for each of the four chromogens, and then unmix the signals of multispectral images. Staining intensity as a measure of FABP4 expression was quantified by the optical density of the respective chromogen per unit area in pixels. Cells positive for FABP4 were counted with colocalization analysis using Inform TM 2.1 software. The following cores were excluded from analysis: a) less than 5% epithelial component, b) significant tissue loss or folding, d) images with more than 5% poorly segmented nuclei. The histological‐score (H‐score) was used to evaluate the staining intensity. For each observed tissue component, a summary value we refer to as H‐Score was calculated. This consists of a sum of the percentages of positively stained cells multiplied by a weighted intensity of staining H‐score = ΣPi(i + 1), where “Pi” is referring to the percentage of stained cells in each intensity category, and “i” is the intensity for i = 1, 2, 3. A total H‐Score for the tissue section was derived as the sum of the component H‐Scores weighted by the fraction of each component observed in the tissue section. The H‐score results were checked in by two experienced researchers, were averaged in the last step. The average H‐score of the tumor tissues were taken as the cut‐off value, which is for the further analysis.

### Cell culture and transfection

Liver cancer cell lines 7404, YY‐8103, MHCC‐97H, and MHCC‐97L were purchased from Cell Bank of Type Culture Collection of Chinese Academy of Sciences. Lentivirual constructs of FABP4 and shFABP4 were constructed using the standard protocols. YY‐8103 and 7404 cells were infected with p23‐ZsGreen‐FABP4 lentivirual, MHCC‐97H, and MHCC‐97L cells were infected with pLKO.1‐shRNA. Overexpressed and silenced cells were sorted using flow cytometry or selected by puromycin (4 μg/mL) for at least 4 days, respectively. All cells were routinely cultured in DMEM (Invitrogen) supplemented with 10% fetal bovine serum (FBS; Life Technologies, New York, USA) in a humidified incubator containing 5% CO2 at 37°C.

### Western blot

Total cell lines protein was extracted using RIPA Lysis Buffer and PMSF (Thermo Scientific) according to the manufacturers’ instructions, after centrifuged at 13,000 g for 15 min, extracted the supernatant for further study. Western blots were performed using specific FABP4 polyclonal antibody (Abcam, ab13979, 1:500) and the GAPDH specific polyclonal antibody (KC‐5G4; Kangcheng Shanghai, China) and Snail, STAT3 and pSTAT3 antibody (Abcam). Secondary antibodies and CA were purchased from Cell Signaling Technology. The images were captured using the Gel Dox XR system (Bio‐Rad, Philadelphia, PA).

### Cell proliferation analysis

Cell proliferation was detected with crystal violet assay or MTT staining method that described by manufacturers’ instructions. Briefly, in crystal violet assay, 1 × 10^3^ cells were seeded into 6‐well plates, the cells were cultured in medium with 10% FBS, changed the medium every three days, 2 weeks later, cell will be stained with crystal violet, after we took the photo, we dissolved it in 2 mL PBS, and the absorbance was measured at a wavelength of 490 nm using a Synergy H4 Hybrid microplate reader (BioTek Inc., Winooski, VT). In MTT assay, 1 × 10^3^ cells were seeded into 96‐well plates, and cell viability was detected by MTT, after 1, 2, 3, 4, 5 or 6 day incubation, 20 μL of a 5 mg/mL MTT solution was added to each well, the plate was continued to incubate at 37°C for 4 h. Thereafter the medium was aspirated and the wells washed with PBS, drained for approximately 2 h and then the solution was carefully aspirated and 200 μL DMSO was added to dissolve the crystal. The microtitre plate was placed on a shaker to dissolve the dye, the absorbance was measured at a wavelength of 570 nm.

### Boyden chamber assay

2 × 10^5^ cells were added to the upper chamber (without Matrigel). Medium with fetal bovine serum was loaded into bottom wells to induce migration and invasion. Cells incubated for 4–6 h and stained with hematoxylin and eosin (Boyden chamber). Randomly selected five fields and counted cells.

### Follow‐up

All patients reexamined serum AFP levels, liver function, and abdominal USG once a month. Chest X‐rays and contrast CT scan were performed every 3 months for monitoring recurring. MRI or PET‐CT was performed in patients with suspected recurrence or metastases. The overall survival (OS) was defined as the interval between surgery and the date the patient died. Recurrence‐free survival (RFS) was defined as the interval between surgery and the date of recurrence. The OS and RFS were censored at the last follow‐up visit (August 31, 2012) for surviving patients and those without recurrence. Micro‐metastases were defined as tumors adjacent to the border of the main tumor that could only be observed under a microscope.

### Tumorigenesis in vivo

Suspended cells (2 × 10^6^) were injected into 5‐week‐old male nude mice treated in accordance with AAALAC criterion. Each animal was subcutaneously injected at two sites in the flanks. Tumor volume (cm^3^) was measured every 7 days from day 7 and tumor weight was measured at last.

### Statistical analysis

All statistical analyses were performed using SPSS 19.0 statistical software (SPSS Inc., Chicago, IL). A chi‐square test was used to evaluate the association between the FABP4 expression level and the clinicopathological characteristics. We used Kaplan–Meier method to evaluate the cumulative survival, and the significance of the differences was using the log‐rank test. Furthermore, we used Cox multivariate regression analysis to determine the independent prognostic factors of HCC. The statistical results were considered significant at *P *< 0.05 and highly significant at *P *< 0.01.

## Results

### Expression of FABP4 is down‐regulated in HCC tissue samples

**Figure 1:**
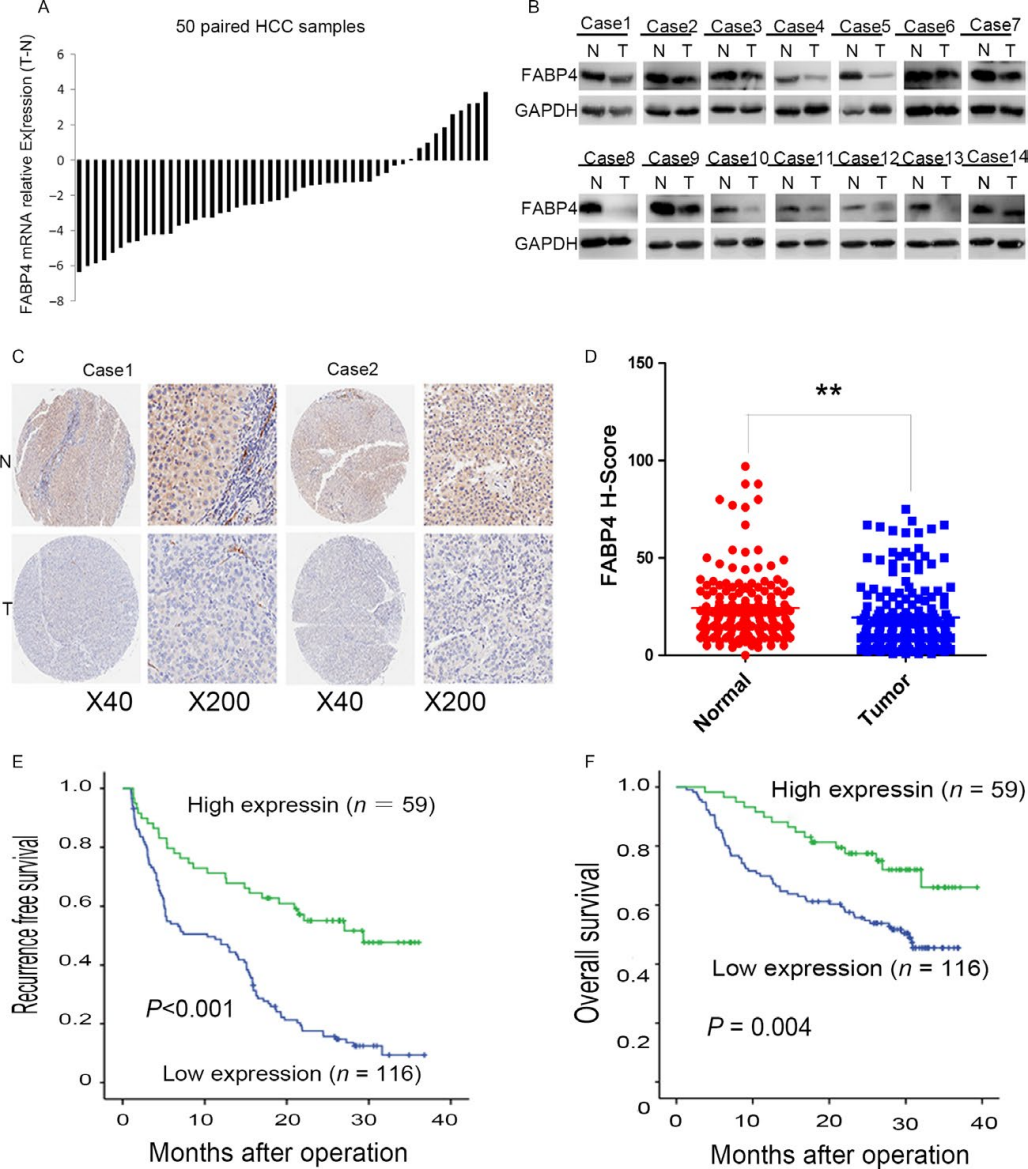
FABP4 expression was increased in HCC tissues and correlated with the survival of HCC patients. (A) The mRNA level of FABP4 was examined by q‐PCR in 50 pairs of tumor samples (T) and matched normal hepatic tissues (N). (B) The protein expression level of FABP4 by Western blot in 14 pairs of N and T from HCC patients. (C) Immunohistochemistry staining of FABP4 in paired N and T from two cases. (D) Scatter plots reflecting the intensity of FABP4 staining in N (*n *= 175) and T (*n *= 175), **, P < 0.01. (E) The high FABP4 expression correlated with better recurrence‐free survival of HCC patients (FABP4 high, *n *= 59; FABP4 low, *n *= 116). (F) The high FABP4 expression correlated with worse overall survival of HCC patients (FABP4 high, *n *= 59; FABP4 low, *n *= 116), *P* = 0.004.

Firstly, we examined the mRNA level of FABP4 in 50 pairs of HCC tissues and the corresponding normal liver tissue, and we found that the mRNA expression of FABP4 was significantly decreased in 30 of 50 HCC tissues compared to the paired normal liver tissues (Fig. 1A). We also examined expression of FABP4 at protein level by Western blot. As shown in Figure 1B, FABP4 expression was dramatically reduced in most of HCC tissues when compared to their normal counterparts. To further confirm our observation, a set of tissue microarray was employed, and the expression of FABP4 was examined in 175 pairs of HCC tissues using immunohistochemistry. We found that FABP4 was mainly expressed in cytoplasm, and the tumor tissues showed weaker staining when compared to the paired normal tissues (Fig. 1C), which was consistent with the results of real‐time PCR and Western blot.

### Clinical significance of FABP4 in HCC patients

To further explore the clinical significance of FABP4 in HCC, the relationship between FABP4 and clinicopathological characteristics was analyzed. All patients were divided into two groups according to IHC staining intensity (H‐score) of FABP4 in tumor tissues. We found that low FABP4 expression was significantly correlated with Alb (*P *= 0.038), AFP level (*P *= 0.007), CA19‐9 (*P *= 0.048), PVTT (*P* = 0.016), tumor size (*P* = 0.018), and encapsulation (*P* = 0.022). And there was no significant correlation between FABP4 and other clinicopathological variables, including gender, age, AST, PT, HBsAg, number of tumor, MVI, cell differentiation, and BCLC (Table 1).

**Table 1:** Correlation between the expression of FABP4 and clinicopathological characteristics

Variables	Number	FABP4, *n* (%)		*P*‐value
		Low expression	High expression	
Gender				
Male	159	102 (87.93)	57 (96.61)	0.093
Female	16	14 (12.07)	2 (3.39)	
Age, year				
<50	97	66 (56.90)	31 (52.54)	0.584
≥50	78	50 (43.10)	28 (47.46)	
AFP, μg/L				
<20	54	28 (24.14)	26 (44.07)	**0.007**
≥20	121	88 (75.86)	33 (55.93)	
PVTT				
Negative	148	93 (80.2)	55 (93.2)	**0.016**
Positive	27	23 (19.8)	4 (6.7)	
Tumor size, cm				
<5	82	47 (40.52)	35 (59.32)	**0.018**
≥5	93	69 (59.48)	24 (40.68)	
Number of tumor				
Single	155	105 (90.52)	50 (84.75)	0.566
Multi	20	11 (9.48)	9 (15.25)	
Tumor encapsulation				
No	123	75 (64.66)	48 (81.36)	**0.022**
Yes	52	41 (35.34)	11 (18.64)	
MVI				
Negative	103	67 (57.76)	36 (61.02)	0.679
Positive	72	49 (42.24)	23 (38.98)	

P < 0.05 is showed in bold.

We also examined the impact of FABP4 expression on the prognosis of 175 HCC patients after surgery. The recurrence‐free survival (RFS) rate and overall survival (OS) rate were significantly better in FABP4‐high group compared to FABP4‐low group (*P* < 0.001, *P* = 0.004, Fig. 1D and E).

The univariate analysis revealed that the AST, AFP, GGT, ALP, PVTT, tumor size, micro metastasis, encapsulation, MVI, BCLC, and FABP4 expression were correlated with RFS of HCC patients (Table 2). And we the Alb, AST, AFP, GGT, HBsAg, PVTT, number of tumor, micro metastasis, encapsulation, MVI, BCLC, FABP4 expression were correlated with OS of HCC patients (Table 3). Furthermore, multivariate Cox regression analysis revealed that the FABP4 expression could serve as an independent risk factor for both RFS (Table 2) and OS (Table 3) of HCC patients.

**Table 2:** Univariate and multivariate analysis associated with disease‐free survival

Variables	Univariate		Multivariate		
	HR (95% CI)	*P*‐value	HR	95% CI	*P*‐value
Male sex	0.991 (0.546–1.799)	0.997			
Age ≥50, year	0.761 (0.535–1.082)	0.128			
Alb ≥40, mg/L	0.719 (0.497–1.040)	0.08			
AST ≥40, U/L	1.874 (1.320–2.661)	**<0.001**			0.147
PT ≥12, s	1.144 (0.804–1.630)	0.454			
AFP ≥20, μg/L	2.017 (1.344–3.026)	**0.001**	2.047	1.349–3.106	**0.001**
CA199 ≥ 39, U/mL	1.407 (0.908–2.182)	0.127			
GGT ≥60, U/L	2.282 (1.566–3.326)	**<0.001**	1.989	1.310–3.022	**0.001**
ALP ≥125, U/L	1.584 (1.080–2.325)	**0.019**			0.835
HBsAg, Positive	1.267 (0.619–2.594)	0.518			
PVTT, Positive	2.914 (1.823–4.658)	**<0.001**			0.499
Tumor size, cm	2.149 (1.446–3.194)	**<0.001**	1.221	1.072–1.392	**0.003**
Num of tumor, Multi	1.045 (0.704–1.549)	0.828			
Micro metastasis, Positive	2.027 (1.415–2.902)	**<0.001**			0.186
Encapsulation, Negative	1.617 (1.312–1.993)	**<0.001**	1.595	1.283–1.983	**<0.001**
MVI, Positive	2.03 (1.429–2.884)	**<0.001**			0.446
BCLC, 0, A, B, C, D	2.184 (1.684–2.834)	**<0.001**			0.586
FABP4 expression, High	0.351 (0.230–0.536)	**<0.001**	0.439	0.285–0.675	**<0.001**

P < 0.05 is showed in bold.

**Table 3:** Univariate and multivariate analysis associated with overall survival

Variables	Univariate		Multivariate		
	HR (95% CI)	*P*‐value	HR	95% CI	*P*‐value
Gender, male	0.852 (0.370–1.963)	0.708			
Age ≥50, year	0.906 (0.573–1.432)	0.672			
Alb ≥40, mg/L	0.597 (0.374–0.954)	**0.031**	0.547	0.333–0.899	**0.017**
AST ≥40, U/L	2.041 (1.291–3.227)	**0.002**			0.167
PT ≥12, s	1.134 (0.713–1.801)	0.596			
AFP ≥20, μg/L	2.717 (1.494–4.944)	**0.001**	2.37	1.291–4.351	**0.005**
CA199 ≥ 39, U/mL	1.718 (1.068–3.091)	0.028			0.220
GGT ≥60, U/L	3.253 (1.869–5.663)	**<0.001**			0.301
ALP ≥125, U/L	1.873 (0.590–5.948)	0.287			
HBsAg, Positive	4.096 (2.414–6.950)	**<0.001**	3.289	1.870–5.784	**<0.001**
PVTT, Positive	2.826 (1.578–5.062)	**<0.001**	1.132	1.068–1.199	**<0.001**
Tumor size, cm	1.132 (0.704–1.821)	0.608			
Num of tumor, Multi	2.218 (1.405–3.500)	**0.001**			0.119
Micro metastasis, Positive	1.381 (1.043–1.8 27)	**0.024**			0.147
Encapsulation, Negative	1.786 (1.355–2.355)	**<0.001**			0.066
MVI, Positive	1.807 (1.147–2.845)	**0.011**			0.750
BCLC, 0, A, B, C, D	1.682 (1.364–2.075)	**<0.001**			0.146
FABP4 expression, High	0.45 (0.334–0.916)	**0.004**	0.545	0.310–0.958	**0.035**

P < 0.05 is showed in bold.

### Overexpression of FABP4 inhibited proliferation and migration of HCC cell lines

**Figure 2:**
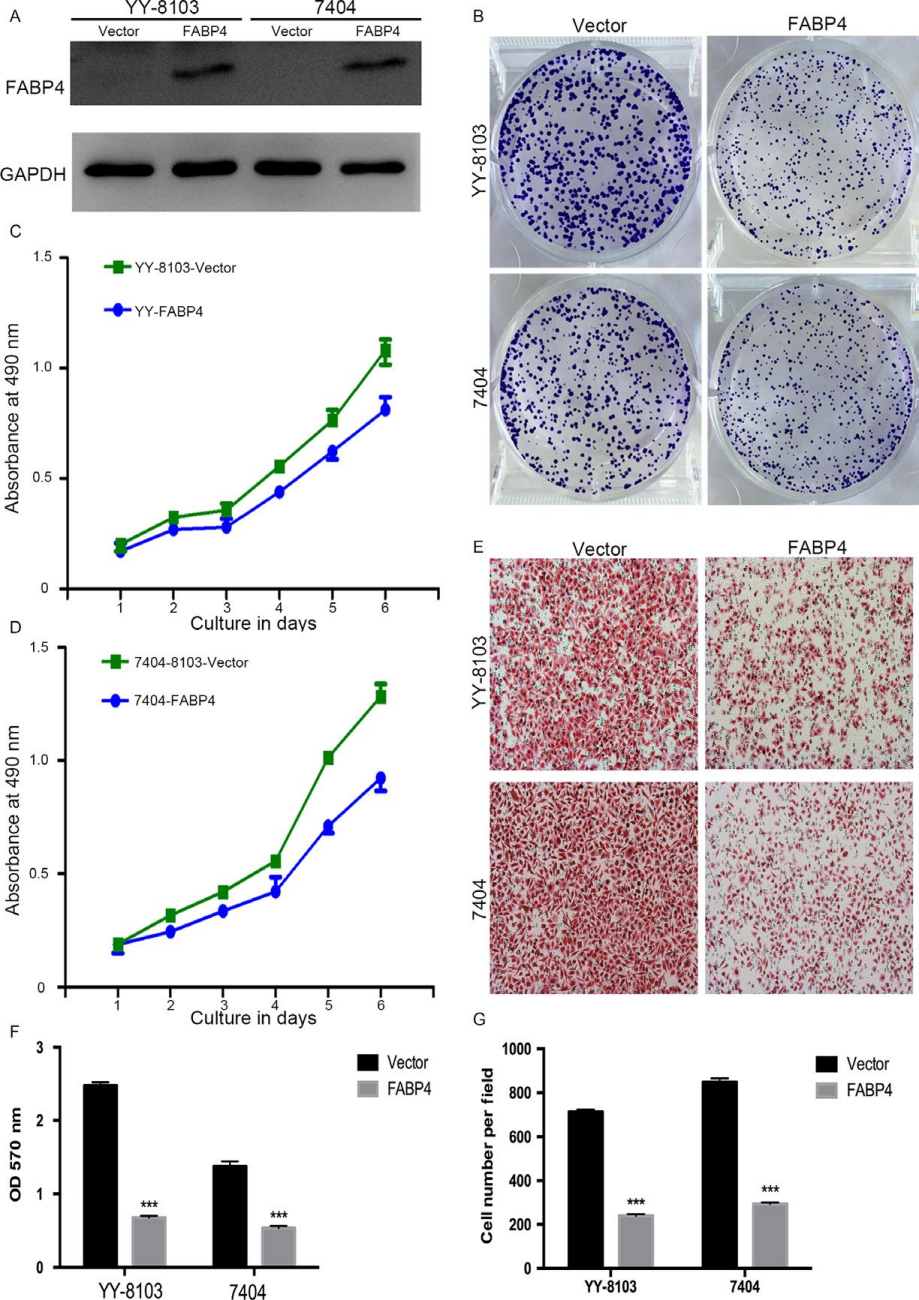
FABP4 overexpression inhibited growth, colony formation, and migration of HCC cells in vitro. (A) Western blot examined the overexpression of FABP4 in YY‐8103 and 7404. (B) Effects of FABP4 overexpression on viability of YY‐8103 and 7404 cells were assessed by crystal violet assay. (C) Effects of FABP4 overexpression on viability of YY‐8103 cells were assessed by MTT. (D) Effects of FABP4 overexpression on viability of 7404 cells were assessed by MTT. (E) Effects of FABP4 overexpression on migration of YY‐8103 and 7404 cells were assessed by Boyden chamber assay. (F, G) statistic results of crystal violet assay and Boyden chamber assay. Data were presented as the mean ± SEM.

The above clinical studies suggested that FABP4 was probably a tumor suppressor in HCC. To identify this hypothesis, FABP4 was either forced expressed or knocked down by RNAi in HCC cells. To evaluate the functional of FABP4 in vitro, we overexpressed FABP4 in YY‐8103 and Bel‐7404 (Fig. 2A) and assessed the effect on proliferation. The crystal violet (Fig. 2B) assays demonstrated that FABP4 modulates the proliferation rate of HCC cells, and MTT (Fig. 2C and D) assay also prompted the same results. The Boyden chamber assay significantly decreased migration of FABP4 overexpression HCC cells (Fig. 2E). Finally, the quantity of crystal violet (Fig. 2F) and Boyden chamber assay (Fig. 2G) had significant difference.

### Low‐expression of FABP4 promoted proliferation and migration of HCC cell lines

**Figure 3:**
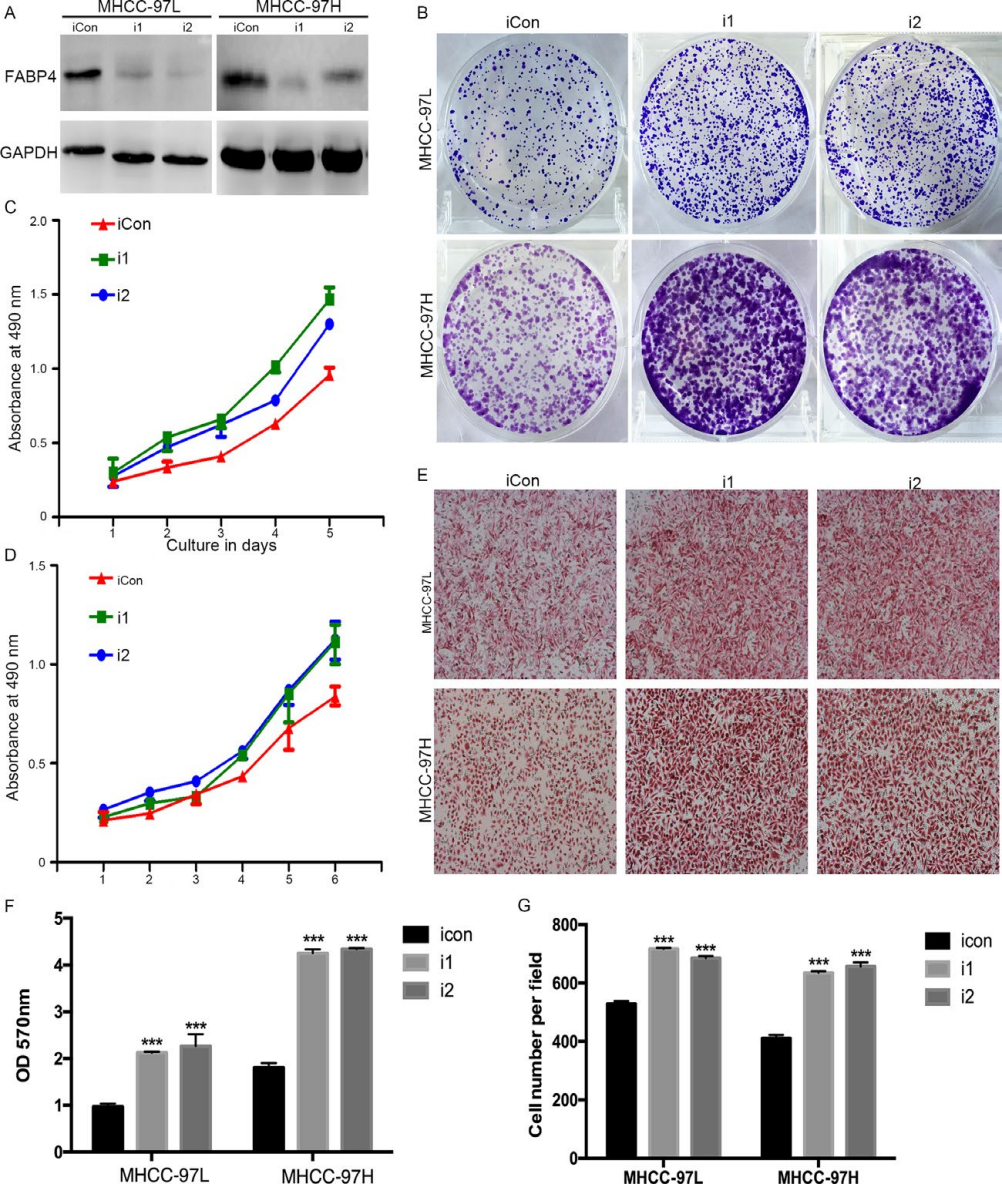
Knockdown of FABP4 expression promoted growth, colony formation, and migration of HCC cells in vitro. (A) Western blot examined knockdown of FABP4 in MHCC‐97L and MHCC‐97H. (B) Effects of FABP4 low expression on viability of MHCC‐97L and MHCC‐97H cells were assessed by crystal violet assay. (C, D) Effects of knockdown of FABP4 expression on viability of MHCC‐97L and MHCC‐97H cells were assessed by MTT. (E) Effects of FABP4 low expression on migration of MHCC‐97L and MHCC‐97H were examined by Boyden chamber assay. (F, G) statistic results of crystal violet assay and Boyden chamber assay. Data were presented as the mean ± SEM. *, *P* < 0.05; **, *P* < 0.01; ***, *P* < 0.001.

We also evaluated the role of FABP4 low‐expression on cell by MTT, crystal violet, and the Boyden chamber experiment in Figure 3. Firstly, we established low‐expression of FABP4 in MHCC‐97H and MHCC‐97L cell (Fig. 3A). The MTT, crystal violet and the Boyden chamber experiment revealed that siRNA‐mediated suppression of FABP4 may promote the proliferation and migration of HCC (Fig. 3B,C,D and E). The quantity of crystal violet (Fig. 3F) and Boyden chamber assay (Fig. 3G) showed the same results.

### FABP4 suppressed tumor development in vivo

**Figure 4:**
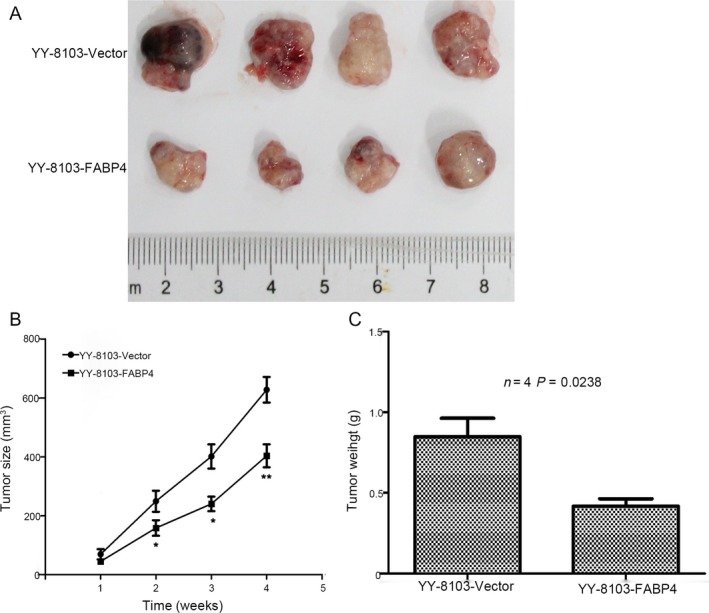
Overexpression of FABP4 inhibited tumor development in vivo. (A) Pictures of tumors generated by controls cells and overexpression of FABP4 of YY‐8103 cells. (B) The growth curve of tumors (cm^3^). (C) The Weights of tumors (g). Each bar represented the mean ± SD. *P < 0.05; **P < 0.01; ***, *P* < 0.001.

To further explored whether FABP4 could suppress the growth of HCC cells in vivo, control, and FABP4‐overexpressing YY‐8103 cells were injected into the subcutaneously of nude mice to monitored the tumor growth (Fig. 4A).The average tumor volume in the FABP4‐overexpressing group was significantly suppressed compared with those in the control group (*n *= 4, *P* < 0.01; Fig. 3B). The tumor weight of FABP4 overexpression was smaller and lighter than those generated by control cells (*n *= 4, *P* = 0.0238; Fig. 4C). It suggested that FABP4 inhibited tumor grow of HCC cells in vivo, and these results were consistent with the inhibited growth of HCC cells by FABP4 in vitro.

### FABP4 induces Snail of epithelial‐to‐mesenchymal transition and P‐Stat3 signaling

**Figure 5:**
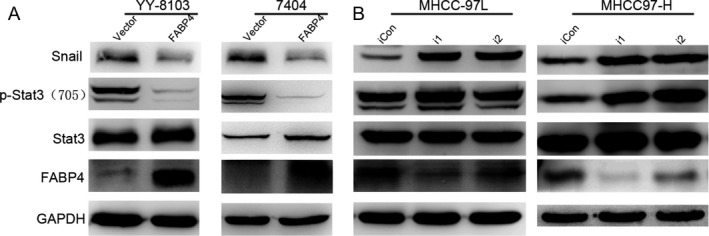
FABP4 induced Snail of epithelial‐to‐mesenchymal transition and STAT3 signaling in HCC cells. (A) Overexpression of FABP4 suppressed the expression of Snail of epithelial‐to‐mesenchymal transition and P‐Stata3 of STAT3 signaling in Bel‐7404 and YY‐8103 by Western blot, respectively. (B) Knockdown of FABP4 activated the Snail of epithelial‐to‐mesenchymal transition and P‐Stat3 of STAT3 signaling in MHCC‐97L and MHCC‐97H by Western blot, respectively. GAPDH was used as a loading control.

To explore the molecular mechanism of FABP4 on HCC, Western blot analysis was performed to investigate the expression levels of Snail of epithelial‐mesenchymal transition markers and Phosphorylation‐Stat3 (p‐Stat3) of STAT3 signaling. Expression levels of Snail and P‐Stat3 were significantly decreased by overexpression of FABP4 (Fig. 5A). However, we observed the opposite effects in MHCC97L and MHCC97H cells. Knockdown of FABP4 may increase the expression levels of Snail and P‐Stat3 that were relevant with tumor progression and metastasis.

## Discussion

Over the past decades, the prognosis of HCC is still poor, and it still lacks a high sensitive and specified diagnosis of early diagnosis of HCC. Among all therapies for HCC, surgical is the main treatment. If HCC can be diagnosed at the early stage, the prognosis of HCC will be greatly improved. However, the previous studies found many tumor markers, but lots of these markers turned out lack of practical clinical value. Under these circumstances, it is urgent to find effective prognostic biomarkers for HCC.

In recent years, the growing evidence proved that FABP family, which contains at least 10 proteins is not only playing a role in intracellular lipid transport and metabolism but also in intracellular signal transduction. Several subtypes of FABP family have been confirmed playing an important role in various cancers. For example, high FABP3 expression promotes gastric carcinoma aggressiveness and poor survival rate, FABP7 is a marker for renal cell carcinomas 20, FABP5 is associated with drug‐resistant in pancreatic cancer and had a significant role for FABP5 in HCC progression and metastasis through the induction of epithelial‐to‐mesenchymal transition 21. FABP4 belongs to FABP family and primarily expressed in macrophages and adipocytes. However, FABP4 can be changed in many kinds of cancers. Previous study indicated that FABP4 regulated the VEGF and promoted the proliferation of HUVEC, and it also reported in various cancers. Boiteux et al. 11 reported that there was a loss of FABP4 expression in poor prognosis high‐grade pT1 bladder tumors compared to good prognosis tumors, further the transcript level significantly correlated to tumor stage and to histologic grade in bladder cancer. Tang et al. 15 reported that expression of FABP4 in non‐small cell lung cancer (NSCLC) was significantly associated with advanced tumor node metastasis (TNM) stage and had a negative impact on the OS of NSCLC patients. However, the function of FABP4 in HCC is still unclear.

In our study, we reported the expression and clinical significance of FABP4 using tissue microarray technology, we found that decreased FABP4 expression was related to poor OS (HR 0.545, 95% CI 0.310–0.958; *P* = 0.035) and RFS (HR 0.439, 95% CI 0.285–0.675; *P* < 0.001). We revealed that FABP4 was significantly low‐expression in HCC tissues compared to the corresponding tissue adjacent at both mRNA and protein level, and the expression level was correlated with tumor size and PVTT. Furthermore, we found that FABP4 inhibit proliferation and migration of HCC cell lines (YY‐8103, 7404, MHCC‐97H, and MHCC‐97L). Therefore, we put forward a hypothesis that FABP4 plays an important role in the occurrence of HCC development and will be a potential target for HCC therapy. To prove our hypothesis, with stable clones of YY‐8103‐FABP4, 7404‐FABP4, si‐MHCC‐97H, and si‐MHCC‐97L cells exhibiting inhibited the proliferation and migration relative to control groups. In vivo, we confirmed that FABP4 suppressed tumor development in YY‐8103 cells. Meanwhile, FABP4 significantly suppressed growth and migration of HCC cells possibly through regulation of Snail and p‐STAT3 signaling. The further study is needed to explore the tumor‐specific function of FABP4.

Moreover, as for the mechanism of FABP4 in other various cancers 17, many studies have been reported. Jin et al. 22 have reported that FABP4 promotes epithelial‐mesenchymal transition in cervical squamous cell carcinoma through AKT/GSK3β/Snail signaling pathway. Jin et al. 23 have suggested that FABP4 has relation with mTOR pathway. Stat3 is a well‐known tumor helper in kinds of cancers. Won et al. have reported that TLR9 expression and secretion of LIF by prostate cancer cells stimulates accumulation and activity of polymorphonuclear MDSCs through stat3 pathway. Ai et al. 24 have reported that 20(S)‐25‐methoxyl‐dammarane‐3β, 12β, 20‐triol negatively regulates activation of STAT3 and ERK pathways and exhibits anti‐cancer effects in HepG2 cells. But the relation of FABP4 and Stat3 pathway has not been reported. Our present study showed that FABP4 can suppress the phosphorylation of stat3 through Ras‐p‐Stat3signaling pathway and finally lead to the decreasing of HCC.

Taken together, these results indicate that FABP4 low‐expression plays a critical role in the proliferation and metastasis of HCC cells, and maybe a biomarker for HCC diagnosis and prognosis, and may provide an emerging therapeutic target for HCC in future.

## Conflict of Interest

The authors who have taken part in this study declared that they do not have anything to disclose regarding funding or conflict of interests with respect to this manuscript.
